# A surprising finding after adenosine administration

**DOI:** 10.1007/s12471-017-0993-0

**Published:** 2017-04-26

**Authors:** C. Timmermans, H. Wellens

**Affiliations:** 1 Department of Cardiology, Maastricht University Medical Center, Maastricht, The Netherlands; 2 21 Henric van Veldekeplein, Maastricht, The Netherlands

The patient was a 22-year-old man with a 8-year history of episodes of a rapid heart rhythm. A 12-lead ECG during sinus rhythm (not shown) was normal. The ECG of Fig. 1 was recorded in the catheterisation room after adenosine administration and shows sinus rate slowing, changes in QRS configuration and an episode of complete AV block. Thereafter AV conduction resumes.

**Fig. 1:**
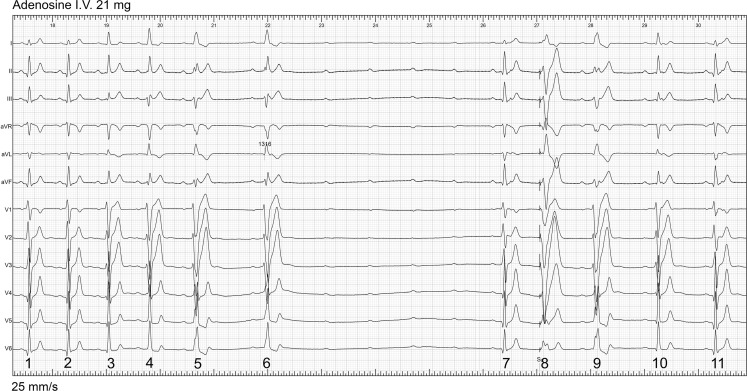
Twelve-lead ECG after adenosine administration during sinus rhythm

Can you explain the QRS changes in beats 3 to 6, and those in beats 7 to 11?

## Answer

You will find the answer elsewhere in this issue.

